# Successful treatment with rituximab of a type 2 diabetes patient with antibody-mediated insulin resistance syndrome: A case report

**DOI:** 10.1097/MD.0000000000043501

**Published:** 2025-07-18

**Authors:** Ramazan Çakmak, Sakin Tekin, Hülya Hacişahinoğullari, Ümmü Mutlu, Özge Telci Çaklili, Vefa Seferova Nasibopva, Ayşe Merve Ok, Göktuğ Saribeyliler, Mustafa Araz, Gülşah Yenidünya Yalin, Özlem Soyluk Selçukbiricik, Nurdan Gül, Ayşe Kubat Üzüm, Kubilay Karşidağ, Nevin Dinççağ, Mehmet Temel Yilmaz, İlhan Satman

**Affiliations:** a Division of Endocrinology and Metabolism Disease, Department of Internal Medicine, Istanbul Faculty of Medicine, Istanbul University, Istanbul, Turkey; b Division of Endocrinology and Metabolism Disease, Department of Internal Medicine, Gaziantep University, Gaziantep, Turkey.

**Keywords:** exogenous insulin antibody syndrome (EIAS), insulin autoimmune syndrome (IAS), insulin resistance, rituximab, type 2 diabetes mellitus

## Abstract

**Rationale:**

Insulin antibody-mediated insulin resistance is a rare autoimmune mechanism that can cause severe hyperglycemia.

**Patient concerns:**

A 52-year-old male patient was admitted to our hospital with complaints of polydipsia, polyuria, and weight loss (8 kg in 6 months). He was diagnosed with type 2 diabetes mellitus at age 33.

**Diagnoses:**

Type 2 diabetes mellitus with antibody-mediated insulin resistance syndrome.

**Interventions:**

Rituximab infusion in a 500 mg dose was given 2 times in a 2-week interval.

**Outcomes:**

A significant response was achieved 1 month later with fasting plasma glucose: 120 mg/dL (6.7 mmol/L), glycosylated hemoglobin A1c: 7.6% (59.6 mmol/mol), and anti-insulin antibodies: 0.001 U/mL. Clinically improved response persisted for about 6 months.

**Lessons:**

Clinicians should be aware of the antibody-mediated insulin resistance, recognize suggestive signs and symptoms, pursue appropriate diagnostic evaluation, and treatment approach.

## 1. Introduction

Although insulin resistance plays a key role in the pathogenesis of type 2 diabetes mellitus (T2DM), clinical cases of insulin resistance in which hyperglycemia remains uncontrolled despite a total insulin daily requirement exceeding 200 IU (or >2 IU/kg) represent rare syndromes.^[1]^ Insulin resistance syndromes (IRS) are conventionally divided into 2 groups: genetic (type A) and immunologic (type B). While in type A IRS, heterozygous mutations in the insulin receptor (INSR) gene, in type B IRS, autoantibodies against INSR are responsible. The clinical picture is characterized by hyperinsulinemia, hypoglycemia, acanthosis nigricans, hypertrichosis, and polycystic ovary with high morbidity and mortality.^[2]^ Dysglycemia may range from normal glucose tolerance to severe insulin resistance refractory to large doses of exogenous insulin. Type B IRS has been reported more frequently in women over 60 years of age, in those with concomitant autoimmune diseases such as systemic lupus erythematosus, and in Japanese.^[3,4]^

However, autoantibodies against insulin itself (insulin autoantibodies: IAA), which develop spontaneously on a genetic background or during certain autoimmune and infectious diseases or due to medications containing sulfhydryl group, may lead to a clinical picture similar to type B IRS, named as Hirata’s disease or insulin autoimmune syndrome (IAS). IAS pathogenesis involves the formation of insulin-IAA complexes, inducing first a mild hyperglycemia in the postprandial period, followed by hypoglycemia. IAS is generally considered a self-remitting disease. It has also been known that antibodies against exogenous insulin (anti-insulin antibodies: AIA) develop in nearly half of patients with T2DM, especially during the period when animal insulins were used.^[5]^ Although AIAs have been considerably reduced after the use of recombinant human insulin and highly purified insulin analogs, AIAs can still be developed with new insulins.^[6]^ Generally, AIAs are accepted that they do not cause a very serious condition clinically and do not disrupt glycemic control.^[7]^ But rarely, immune complexes formed due to insulin-binding properties can lead to high blood glucose levels and serious insulin resistance despite very high insulin requirements. This clinical condition is called “exogenous insulin antibody syndrome (EIAS)” or “antibody-mediated IRS.” The diagnosis of EIAS is based on excluding other causes of insulin resistance and the detection of AIAs in blood samples. There is no specific treatment for EIAS. Similar to the type B IRS, various treatment approaches such as changing insulin preparations, cessation of insulin treatment, adding oral antidiabetics, corticosteroids, intravenous immunoglobulin, plasmapheresis, cyclosporine, azathioprine, and combinations of these have been tried.^[3,8,9]^

In recent years, reports have been published showing that cases of type B IRS have been treated with rituximab.^[10]^ However, there is little to no experience on the effectiveness of rituximab in EIAS cases.^[11]^ Herein, we reported a patient with EIAS who needed extremely high insulin doses and was successfully treated with rituximab.

## 2. Case report

A 52-year-old male patient was admitted to the endocrinology clinic with polydipsia, polyuria, and weight loss (8 kg in 6 months). He was diagnosed with T2DM at the age of 33. During the first 3 years, he used metformin 2000 mg/d and a sulfonylurea (gliclazide MR 30 mg/d). Approximately 3.5 years after the diagnosis, the treatment was changed to basal insulin and metformin. He remained on this treatment with reasonable diabetes control for 11 years. Three years ago, he was successfully treated with transurethral resection for a bladder tumor. Immediately after this therapy, glucose levels started to rise abnormally. Biphasic insulin aspart (Novomix30) was given 15 IU×3. A few months later, biphasic insulin was switched to multiple-dose insulin (MDI) therapy with insulin detemir 50 IU×2 and insulin aspart 24 IU×3. Six months ago, exenatide 10 mcg was added to his treatment, but he could not continue due to severe gastrointestinal intolerance.

**Figure 1.**
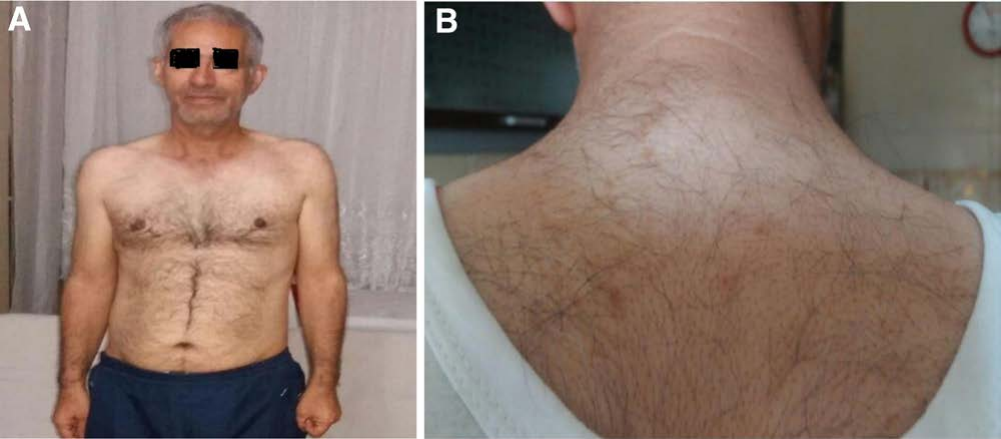
(A, B) Clinical photograph of the patient.

The patient had no history of hypoglycemia. He had hypertension for 7 years and benign prostatic hypertrophy for 4 years. He had a strong family history of diabetes. His mother, 3 sisters, and 1 brother were diagnosed with T2DM at ages 35 to 45, and all had established diabetic complications. On physical examination, body mass index was 25.4 kg/m^2^, blood pressure was 110/80 mm Hg. A mild acanthosis was seen on his neck, but no lipoatrophy, lipohypertrophy or lipodystrophy was detected (Fig. 1). Other system or organ examinations were unremarkable. The patient had symptomatic hyperglycemia despite a very high insulin requirement (2.3 IU/kg/d) and metformin 2000 mg/d. He had also been on perindopril 10 mg/d and doxazosin 4 mg/d therapies for hypertension and benign prostate hypertrophy.

**Figure 2.**
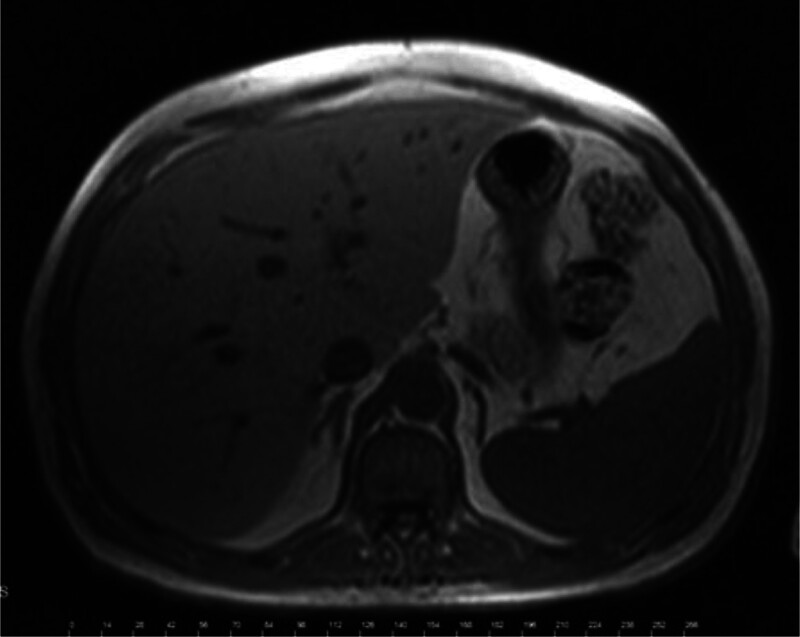
Abdominal MRI scan of the patient (hepatomegaly and hepatosteatosis were seen with normal adrenal glands and pancreas). MRI = magnetic resonance image.

**Figure 3.**
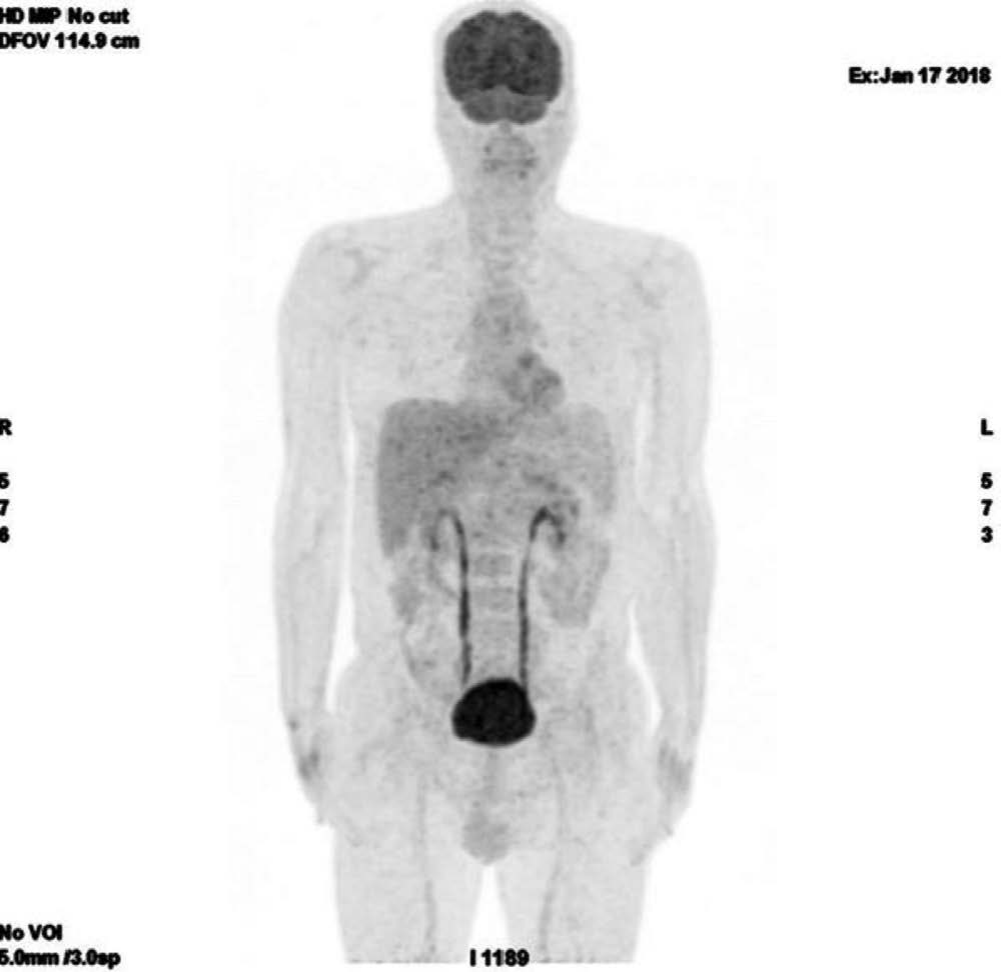
A PET-CT scan showed no primary malignancy or a reactivation of the bladder tumor. PET-CT = positron emission tomography-computed tomography.

Biochemical evaluation revealed fasting plasma glucose (FPG): 492 to 560 mg/dL (27.3–31.1 mmol/L), glycosylated hemoglobin A1c (HbA1c): 13.4% (123 mmol/mol), fasting serum C-peptide: 3.86 ng/mL (1.29 nmol/L; Table 1). Screening for diabetic complications revealed bilateral distal sensory-motor neuropathy and prolonged gastric emptying time (*t*_1/2_: 120 minutes). He had no retinopathy or nephropathy. An abdominal magnetic resonance image revealed severe hepatic steatosis, and hepatomegaly, with normal appearance of the pancreas and adrenal glands (Fig. 2). A positron emission tomography-computed tomography scan was negative for possible primary malignancies or reactivation of the bladder tumor (Fig. 3).

**Table 1:** Laboratory values of the patient at the presentation.

Laboratory tests	Results (reference values)
Fasting blood glucose, mg/dL	492
HbA1c, %	13.4
C-peptide, pg/mL	3.86
LDL-C, mg/dL	196
HDL-C, mg/dL	22
Total-C, mg/dL	336
Triglyceride, mg/dL	612
Anti-TPO antibody	Negative
Anti-Tg antibody	Negative
Antinuclear antibody	Negative
1 mg DXM suppression test	Normal
Midnight salivary cortisol, mcg/dL	0.0054 (<0.2)
Leptin, ng/mL	6 (6–11)
Chromium, mcg/L	1 (0.7–1)
24-h urinary free cortisol, mcg/d	52 (4.3–176)

Anti-Tg = anti-thyroglobulin antibody, anti-TPO = antithyroid peroxidase autoantibody, DXM = dexamethasone, HbA1c = glycosylated hemoglobin A1c, HDL-C = high-density lipoprotein cholesterol, LDL-C = low-density lipoprotein cholesterol, Total-C = total cholesterol.

Potential causes of insulin resistance were investigated. A 24-hour urinary free cortisol: 52 (N: 4.3–176) mcg/24 h, midnight salivary cortisol: 0.054 (N: <0.2) mcg/dL, plasma cortisol after 1 mg dexamethasone: 0.9 mcg/dL. Autoantibodies against glutamic acid decarboxylase, islet cell cytoplasmic, thyroid peroxidase antibody, anti-thyroglobulin, and gliadin (antigliadin IgA) were negative.

Genetic screening with next generation sequencing for 13 common forms of maturity-onset diabetes of the young revealed no pathogenic mutation. An array comparative genomic hybridization analysis was negative, no pathogenic variation was detected in the INSR gene. However, a probable pathogenic variation causing a heterozygous early stop codon in the propionyl-CoA carboxylase subunit beta gene was detected.

**Figure 4.**
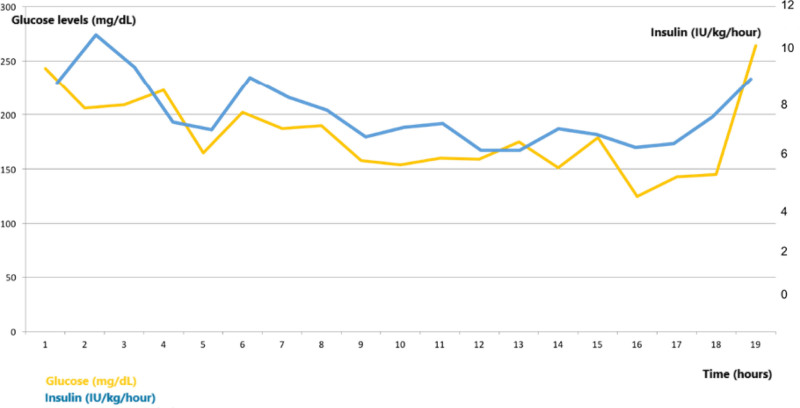
The changes in glucose levels and insulin requirements during insulin infusion treatment.

*Clinical course:* Total daily insulin dose (TDID) was increased up to 320 IU (4.2 IU/kg/d), metformin 2000 mg/d, perindopril 10 mg/d, and escitalopram 10 mg/d. Changing basal insulin to more concentrated forms and preheating the injection sites did not help. Because of the failure of MDI treatment, an intravenous insulin infusion was administered. A partial short-term improvement in glucose levels was achieved, and insulin requirements decreased from 11 IU/h to 7 IU/h with this treatment (Fig. 4). The patient was discharged on his request with the treatment of an insulin pump (with a basal infusion rate of 10 IU/h), insulin aspart 36 IU×3, metformin 2000 mg/d, and dapagliflozin 10 mg/d.

**Figure 5.**
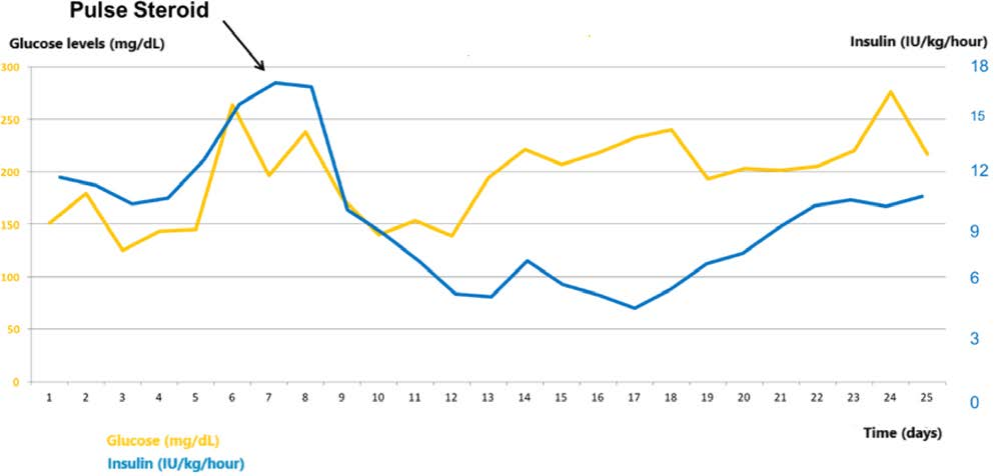
The changes in glucose levels and insulin requirement after glucocorticoid treatment.

After 6 months, he was readmitted to the hospital because of weight loss and poor glycemic control [HbA1c 16% (151.4 mmol/mol)] despite 350 IU of TDID (5.1 IU/kg/d). INSR antibodies were negative (insulin receptor α-chain: 5.7 and β-chain: 5.9, reference range: <10 Ak-ratio); however, AIA was positive for 2 times (5.24 U/mL and 8.43 U/mL; reference: <0.4 U/mL). The patient was diagnosed with antibody-mediated insulin resistance (or EIAS). Glucocorticoid treatment was administered with methylprednisolone (MP) 1000 mg intravenous pulse doses for 5 days, followed by 32 mg/d oral MP and tapered gradually by 4 mg/wk. He also received a preventive isoniazid treatment. The patient was also evaluated for potential coexisting rheumatic diseases, although he denied any signs and symptoms attributed to autoimmune disease. The antinuclear antibody, anti-double stranded deoxyribonucleic acid antibodies, and extractable nuclear antigen panel were negative. Complement levels and inflammatory markers, such as white blood cells, erythrocyte sedimentation rate, and C-reactive protein, were within the normal range. Insulin requirement decreased by 50% within 2 days with this treatment (Fig. 5). The patient was discharged with U300 insulin glargine, 80 IU, insulin aspart 20 IU×3, metformin, and dapagliflozin in the same doses as the previous treatment.

After 4 months, he was readmitted for the third time due to poor glycemic control despite 600 IU of TDID (8.8 IU/kg/d), FPG >400 mg/dL (22.2 mmol/L), HbA1c: 15.2% (143.6 mmol/mol). He developed a background retinopathy. Pulse MP followed by oral MP treatment was restarted with the same protocol, but the beneficial effect of this regimen was limited. Therefore, rituximab infusion in a 500 mg dose was given 2 times in a 2-week interval. Simultaneously, oral MP therapy started and tapered as the previous schema. A significant response was achieved without any side effects 1 month later with FPG: 120 mg/dL (6.7 mmol/L), HbA1c: 7.6% (59.6 mmol/mol), and AIA: 0.001 U/mL. Clinically improved response persisted for about 6 months. One year later, rituximab 500 mg infusion therapy was reordered and repeated in 2 weeks. He was discharged with insulin glargine 27+13 IU, insulin aspart 12+10+8 IU, metformin XR 500 mg/d, pioglitazone 15 mg/d, atorvastatin 20 mg/d, ramipril 5 mg/d, amlodipine 10 mg/d, calcium/vitamin D_3_ 1200 mg/800 IU/d, aspirin 100 mg/d, and sertraline 100 mg/d. The patient has been followed up on an MDI regimen, with a relatively low insulin requirement (0.5–1 IU/kg/d) and favorable glycemic control (HbA1c: 7–8% and FPG: 150–190 mg/dL).

## 3. Discussion

The middle-aged male patient presented here was diagnosed with T2DM at the age of 33. His diabetes was treated with oral antidiabetics metformin and sulfonylurea, for the first 3 years, and insulin was added to his treatment 3.5 years after diagnosis, and acceptable glycemic control was achieved for 11 years. After the first transurethral resection due to bladder tumor 3 years ago, glycemic control suddenly deteriorated, and insulin requirement increased to 2.3 IU/kg/d, despite FPG: 560 mg/dL (31.1 mmol/L), HbA1c: 13.4% (123 mmol/mol) levels. Type 1 diabetes was excluded as endogenous C-peptide level was sufficient, pancreatic islet autoantibodies were negative (even though years had passed since diagnosis), he had not experienced a severe diabetic ketoacidosis, and had no known autoimmunity. Additional endocrinopathies, neuroendocrine tumors, other malignancies, reactivation of bladder tumors, or infections that would increase the patient’s insulin requirement were investigated, and no significant abnormality was detected.

Our patient had insulin resistance as his insulin requirement was over 2 IU/kg/d despite severe hyperglycemia.^[1]^ The patient’s clinic was not compatible with genetic syndromes or lipodystrophies that may lead to insulin resistance. Type A IRS was excluded by not detecting a pathogenic variation in the INSR gene. In addition, due to the intense family history of diabetes, the frequently encountered maturity-onset diabetes of the young forms were investigated with next generation sequencing, and no pathogenic mutation was found.

Then we thought that the patient may have an autoimmune IRS due to autoantibodies against INSR (type B IRS) or exogenous insulin. In the case we presented, the absence of hypoglycemic attacks and hyperinsulinemia, and negative INSR antibodies allowed us to eliminate type B IRS.

It is known that approximately half of the patients with T2DM develop AIA against exogenous insulin. Although it has decreased considerably after the development of highly purified insulins, it is not a very rare situation. As AIA was found to be 20 times higher than the reference value, we thought that the definitive diagnosis of our case is EIAS (or antibody-mediated IRS). Immune complexes formed due to insulin binding properties can lead to high blood glucose levels and serious insulin resistance despite very high insulin requirements. Insulin antibodies can be divided into 2 populations: low-affinity/high-capacity and high-affinity/low-capacity. While the former is commonly found in IAS patients with IAAs, which lead to mild postprandial hyperglycemia and nocturnal hypoglycemia, whereas the latter (high-affinity/low-capacity antibodies) is associated with EIAS, which is typically accompanied by severe insulin resistance.^[11]^ Probably, our case may have high-affinity/low-capacity antibodies to exogenous insulin. However, due to technical limitations, we could not classify AIAs and did not conduct polyethylene glycol test to determine the generation of immune complexes.^[12]^

There is no standardized treatment for EIAS. In the literature, it is reported that treatments such as changing insulin preparations, cessation of insulin treatment, adding various oral antidiabetics, oral methyl prednisolone treatment, pulse MP treatment, intravenous immune globulin, plasmapheresis, cyclosporine, azathioprine, and combinations are used.^[13,14]^ Despite several attempts of changing exogenous insulin preparations, adding pioglitazone and sodium-glucose linked cotransporter inhibitor to the treatment, pre-warming of insulin injection sites, insulin pump application, i.v. insulin infusion, and usage of concentrated insulins, blood glucose control could not be achieved in our patient. In our patient, a relatively good response was obtained to MP pulse and then MP oral treatment, albeit short-term (<6 months). Nevertheless, long-term glucocorticoid use may lead to well-known side effects. Therefore, replacing it with an immunomodulatory treatment is important to prevent potential side effects.

In recent years, reports have been published showing that type B IRS can be treated with rituximab.^[10,15,16]^ Rituximab, a monoclonal antibody developed against CD-20 B-cells, is widely used in several autoimmune syndromes, especially in autoantibody-mediated diseases. Rituximab decreases autoantibody production by depleting B-cells.^[17]^ The efficacy and safety of rituximab as monotherapy were established in several case reports with type B IRS.^[15,16]^

However, there is limited experience on the effectiveness of rituximab in EIAS cases.^[18–20]^ The case we presented is important in terms of showing that rituximab can be used successfully in EIAS cases when necessary. Clinicians should be aware of the autoimmune causes of insulin resistance, recognize suggestive signs and symptoms, and pursue appropriate diagnostic evaluation. Prompt treatment of these cases, including immunomodulators, is key to restoring normoglycemia.

## Author contributions

**Conceptualization:** Ramazan Çakmak, Sakin Tekin, Ümmü Mutlu, Özge Telci Çaklili, Ayşe Merve Ok, Gülşah Yenidünya Yalin, Nurdan Gül, Ayşe Kubat Üzüm, Kubilay Karşidağ, Nevin Dinççağ, Mehmet Temel Yilmaz, İlhan Satman.

**Data curation:** Ramazan Çakmak, Sakin Tekin, Özge Telci Çaklili, Vefa Seferova Nasibopva, Ayşe Merve Ok, Göktuğ Saribeyliler, Mustafa Araz, Kubilay Karşidağ.

**Formal analysis:** Ramazan Çakmak, Hülya Hacişahinoğullari, Özge Telci Çaklili, Vefa Seferova Nasibopva, Göktuğ Saribeyliler.

**Investigation:** Ramazan Çakmak, Hülya Hacişahinoğullari, Ümmü Mutlu, Özge Telci Çaklili, Vefa Seferova Nasibopva, Göktuğ Saribeyliler, Mustafa Araz, Özlem Soyluk Selçukbiricik, Kubilay Karşidağ, Nevin Dinççağ, Mehmet Temel Yilmaz, İlhan Satman.

**Methodology:** Hülya Hacişahinoğullari, Ümmü Mutlu, Özge Telci Çaklili, Vefa Seferova Nasibopva, Göktuğ Saribeyliler, Mustafa Araz, Gülşah Yenidünya Yalin, Mehmet Temel Yilmaz.

**Resources:** Mustafa Araz, Özlem Soyluk Selçukbiricik.

**Supervision:** Ayşe Merve Ok, Gülşah Yenidünya Yalin, Özlem Soyluk Selçukbiricik, Nurdan Gül, Ayşe Kubat Üzüm, Kubilay Karşidağ, Nevin Dinççağ.

**Validation:** Ayşe Merve Ok.

**Writing – original draft:** Ramazan Çakmak, İlhan Satman.

**Writing – review & editing:** Ramazan Çakmak, Sakin Tekin, Hülya Hacişahinoğullari, Ümmü Mutlu, Özge Telci Çaklili, Ayşe Merve Ok, Gülşah Yenidünya Yalin, Özlem Soyluk Selçukbiricik, Nurdan Gül, Ayşe Kubat Üzüm, Nevin Dinççağ, Mehmet Temel Yilmaz, İlhan Satman.
